# Supplementary material for: Giant magneto-photoluminescence at ultralow field in organic microcrystal arrays for on-chip optical magnetometer
Source: Nat Commun. 2024 May 11;15:3995. doi: 10.1038/s41467-024-48464-1 (PMC11088683; doi:10.1038/s41467-024-48464-1)
Supplement: Supplementary file 1 — Supplementary Information [file 41467_2024_48464_MOESM1_ESM.pdf]

Supplementary Information

Giant magneto-photoluminescence at ultralow field in organic microcrystal arrays for on-chip optical magnetometer

Hong Wang[1,2,†], *Baipeng Yin*[1,†], *Junli Bai*[2,3,†], *Xiao Wei*[3,4], *Wenjin Huang*[5], *Qingda Chang*[1,2], *Hao Jia*[1,2], *Rui Chen*[1,2], *Yaxin Zhai*[5], *Yuchen Wu*[2,3,]* *and Chuang Zhang*[1,]*

[1]Key Laboratory of Photochemistry, Beijing National Laboratory for Molecular Sciences, Institute of Chemistry, Chinese Academy of Sciences, Beijing 100190, China

[2]University of Chinese Academy of Sciences, Beijing 100049, China

[3]Key Laboratory of Bio-inspired Materials and Interfacial Science, Technical Institute of Physics and Chemistry, Chinese Academy of Sciences, Beijing 100190, China

[4]Ji Hua Laboratory Foshan, Guangdong 528200, China

[5]Key Laboratory of Low-Dimensional Quantum Structures and Quantum Control of Ministry of Education, Department of Physics, Hunan Normal University, Changsha 410081, China

*e-mail: wuyuchen@iccas.ac.cn; zhangc@iccas.ac.cn.

†These authors contributed equally: Hong Wang, Baipeng Yin, Junli Bai.

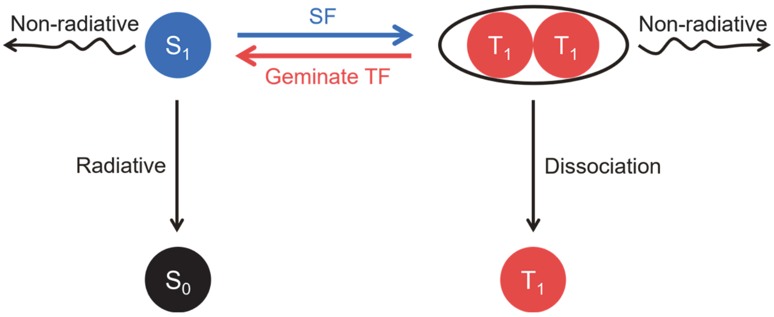

Supplementary Fig. 1 | Spin conversion processes of singlet and triplet in RMCs. The spin conversion processes between geminate singlet fission (SF, blue arrow) and triplet fusion (TF, red arrow) can bridge the different spin state and induce the magneto-photoluminescence effect, while the competition of radiative decay of singlets, the dissociation of triplet-triplet pairs and the non-radiative decay from defects (black arrows) will diminish the magneto-photoluminescence effect.

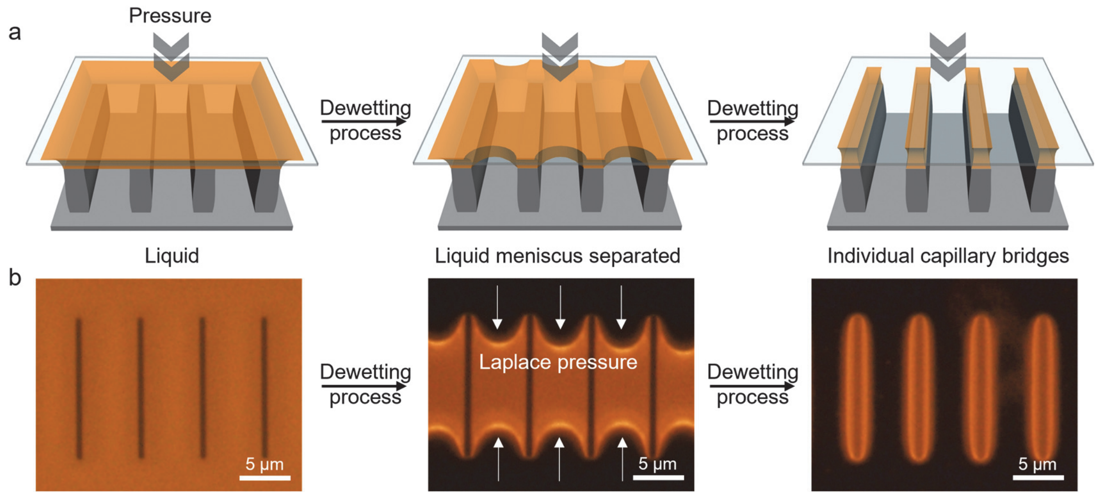

Supplementary Fig. 2 | Fabrication of RMC arrays from the capillary-bridge assembly method. a, Schematic diagram of the liquid bridge dewetting process. **b,** The assembly process of RMCs under microscope. The precursor rubrene solution was carefully dropped onto a micropillar-structured template and then was covered by a Si substrate. The liquid thin film is formed and confined between the target substrate and the micropillar-structure photoresist template under a static pressure. With the evaporation of solvents, the receding of triphase contact line happens at the gaps between two adjacent columns of micropillars driven by the Laplace force. Then the liquid is separated into individual rows and the isolated capillary bridges are anchored onto each individual micropillar. The fluorescence microscopy image of the capillary bridges exhibits uniform fluorescence, indicating the fluid field characteristic and mass transport direction are all the same for each individual capillary bridge. When the rubrene solution becomes supersaturated, the nucleation and growth of rubrene crystals happens in the isolated capillary bridges.

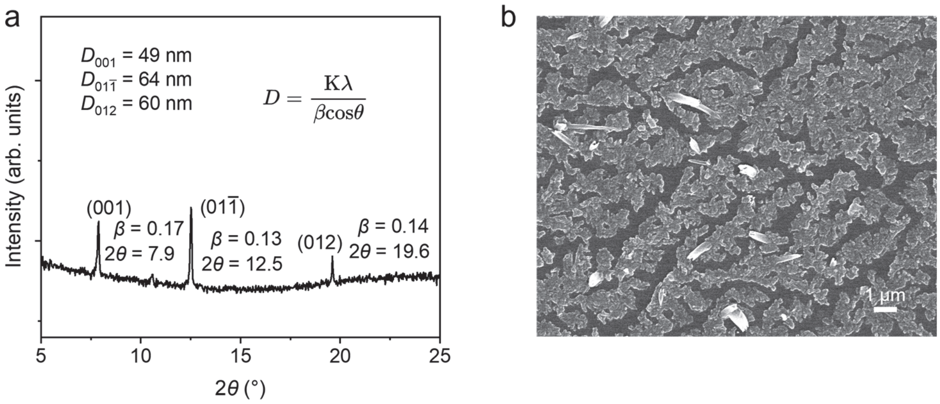

Supplementary Fig. 3 | Crystal grain size in polycrystalline rubrene. a, X-ray diffraction pattern of polycrystalline rubrene film and the calculated grain size. In the equation, D is the calculated particle size, K is the Scherrer constant, λ is wavelength of the X-ray beam, β is the full width at half maximum of the diffraction peak and θ is the Bragg angle. **b,** Zoomed SEM image of polycrystalline rubrene. Source data are provided as a Source Data file.

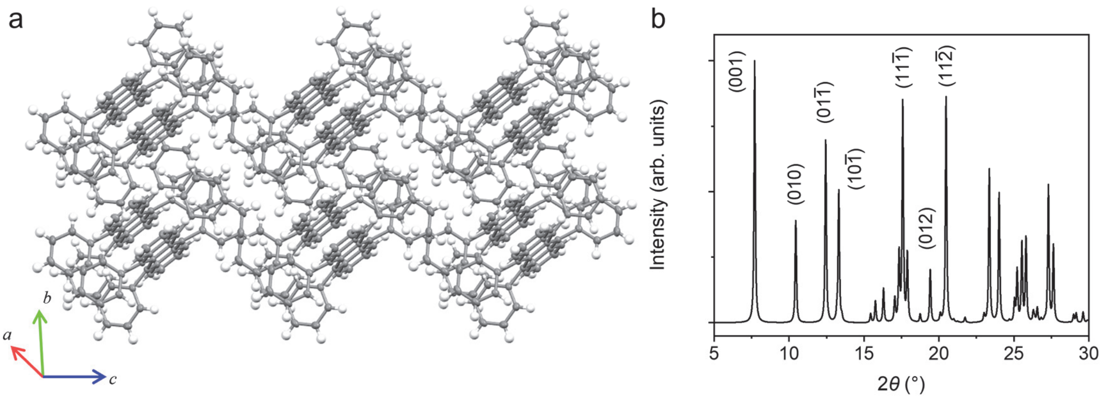

Supplementary Fig. 4 | Crystal characteristics of rubrene in triclinic phase. a, Illustration of the molecular packing in triclinic rubrene. The triclinic crystal exhibits π-stacking interactions between the tetracene backbones which are important for the spin conversion processes of singlet fission and triplet fusion. **b,** The calculated X-ray diffraction patterns of triclinic rubrene powder. Source data are provided as a Source Data file.

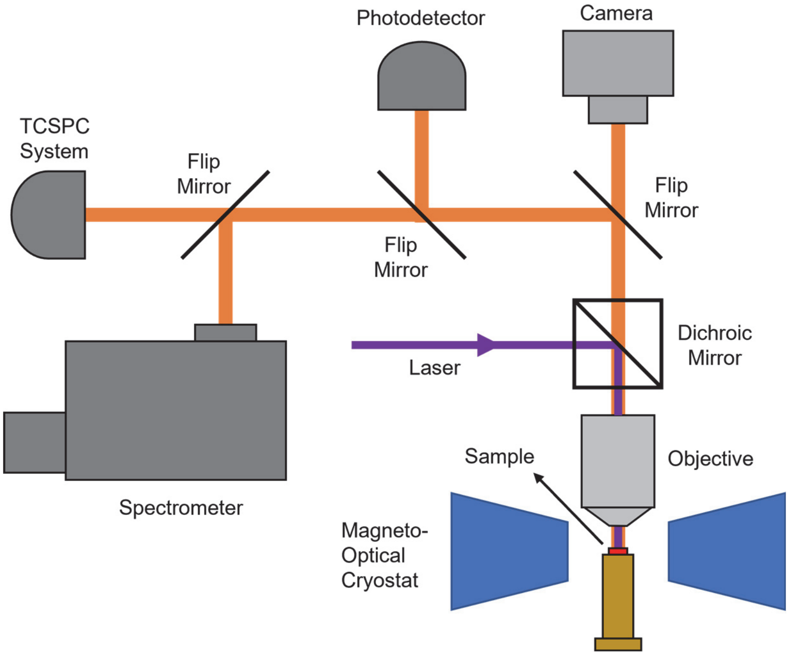

Supplementary Fig. 5 | Schematic of the experiment setup for PL and MPL measurements. The excitation laser was introduced to the RMC samples after a dichroic mirror and a 50× objective. The emitting PL from the samples transmit through the objective and dichroic mirror. The optical pathways can be changed by the flipped mirrors. The camera was used to find the image of RMCs to make sure that the laser can excite the samples accurately.

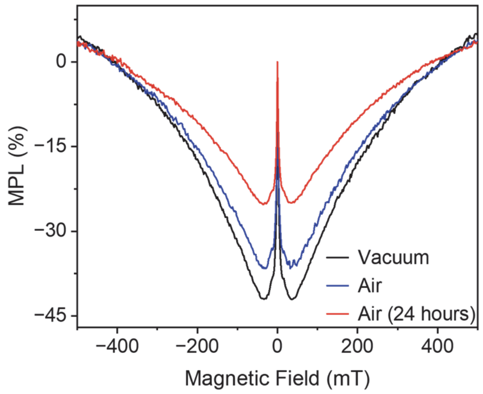

Supplementary Fig. 6 | Influence of oxygen on MPL effect. The MPL(**B**) curves measured from RMC-1.4 μm in vacuum and in the air. As the triplets are sensitive to oxygen, the MPL magnitude slightly decreases immediately after the exposure to air, and it is further reduced to a level of ~25% after a long-time (24 hours) exposure of RMCs in ambient condition. Source data are provided as a Source Data file.

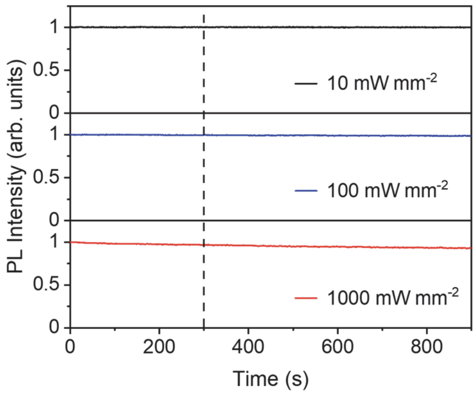

Supplementary Fig. 7 | Stability of PL intensity in RMCs. PL intensity is continuously measured from RMCs under a continuous excitation at different excitation power density of 10, 100, 1000 mW mm^{-2}. In our MPL measurements, the typical excitation power is 100 mW mm^{-2} and it usually takes less than 300 seconds to obtain a typical MPL(**B**) curve. Source data are provided as a Source Data file.

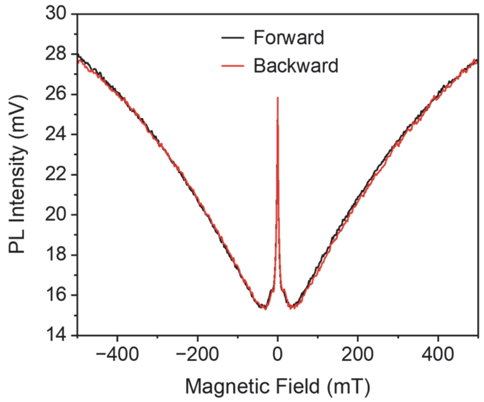

Supplementary Fig. 8 | The contribution of sample degradation to MPL. The forward (-500 mT to +500 mT) scan and backward (+500 mT to -500 mT) scan of MPL(**B**) curves were measured under the excitation power density of 100 mW mm^{-2}. The forward and backward scans are almost identical without any obvious hysteresis. Therefore, the sample degradation should be negligible in the contributions to MPL effects in our experiments. Source data are provided as a Source Data file.

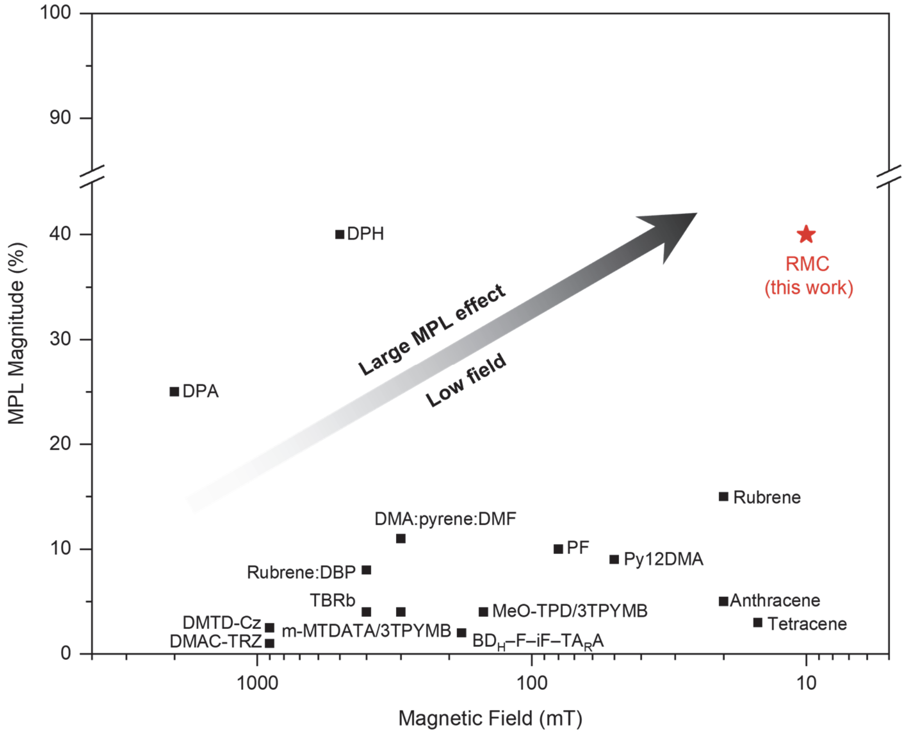

Supplementary Fig. 9 | Summary of the MPL effects observed in organic molecular materials from previous studies[1-15]. Giant MPL effect at low magnetic field can be achieved in RMCs.

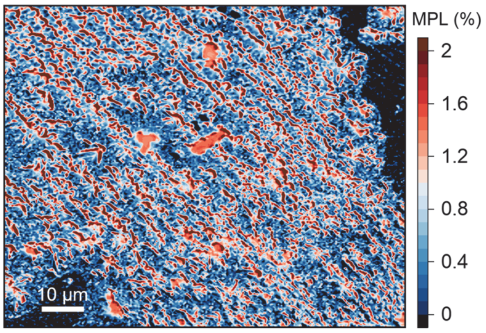

Supplementary Fig. 10 | Spatially resolved MPL of polycrystalline rubrene film. A 50× non-magnetic objective with long work distance was used to measure the luminescence image at zero field and ~10 mT and the spatially resolved MPL image was calculated and averaged from the intensity images converting from original photos. Source data are provided as a Source Data file.

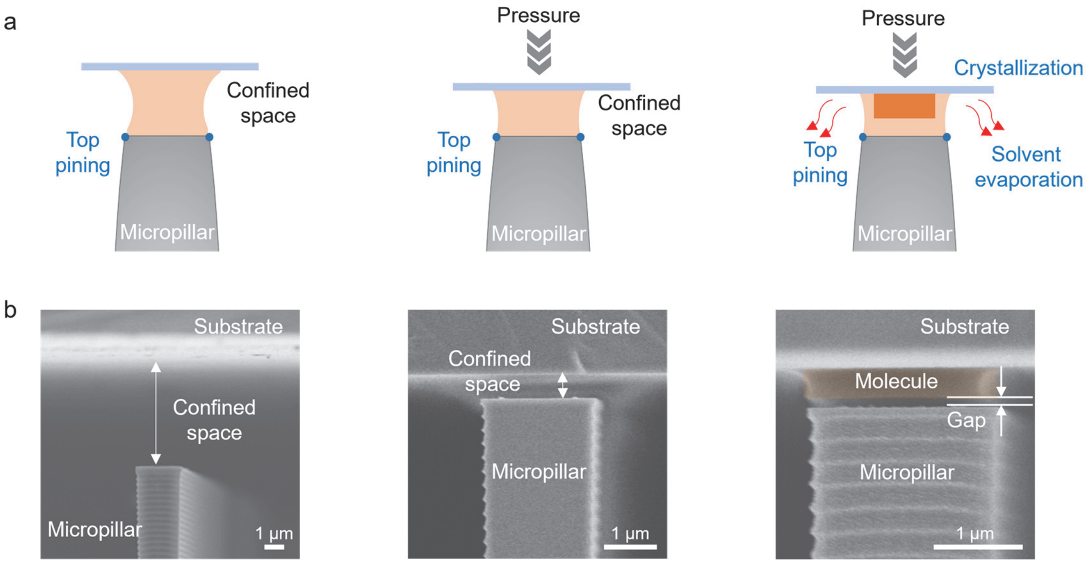

Supplementary Fig. 11 | RMCs growing in confined space with or without pressure. a, Schematic diagram of RMCs nucleus growing in the confined space. **b**, Corresponding environmental scanning electron microscopy images for the cross-sectional view of an individual micropillar with and without pressure. A static pressure was applied in the capillary-bridge assembly to control the size of confined space between the template micropillars and the underneath substrate. The pressure is applied against the surface tension from shape change of liquid film, and the crystals are grown within the liquid film on the substrate. The net pressure on the assembled crystals should be nearly zero, and should not influence the packing of rubrene molecules. Especially, the in-situ scanning electron microscopy image of completely crystallized structures on the right side shows a gap between the assembled crystal the micropillar, which supports that the nucleation/growth processes are always in the liquid environment and unaffected by external pressure.

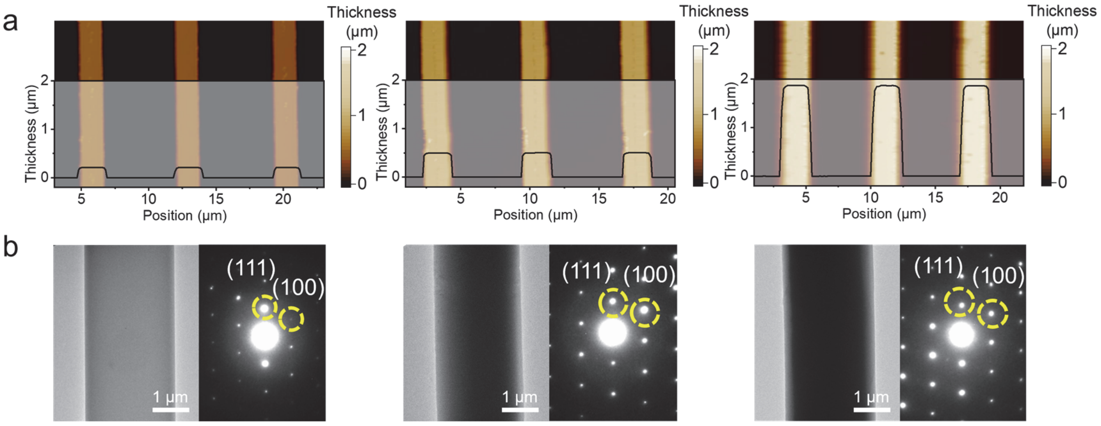

Supplementary Fig. 12 | Molecular packing of the RMCs with different thicknesses. a, The atomic force microscopy images of three adjacent RMCs with the width of ~2 μm but different thicknesses varying from 0.2 μm to 1.9 μm. **b**, The transmission electron microscopy images and selected area electron diffraction patterns of corresponding RMCs with different thicknesses. The molecular packing remains all the same in these samples of RMCs with different thickness. Source data are provided as a Source Data file.

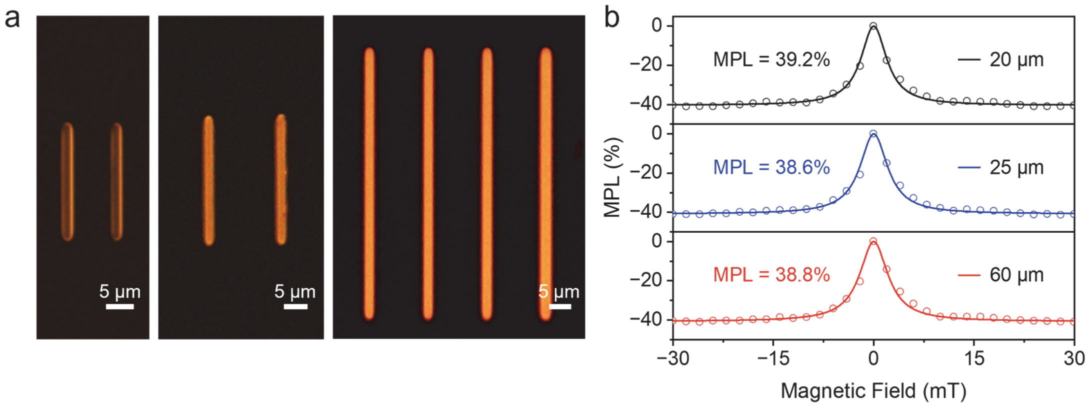

Supplementary Fig. 13 | Length dependent MPL effect in RMC. a, Fluorescence images of RMC-1.4 μm with different lengths. **b,** MPL(**B**) curves measured from corresponding RMCs with different lengths (20, 25 and 60 μm). The lengths of RMCs (with constant thickness of 1.4 μm and width of 2 μm) are tuned from 20 to 60 μm. These samples show very similar MPL responses of ~40% at 10 mT, indicating that MPL effect is not obviously influenced by the lengths of RMCs in our experiments. Source data are provided as a Source Data file.

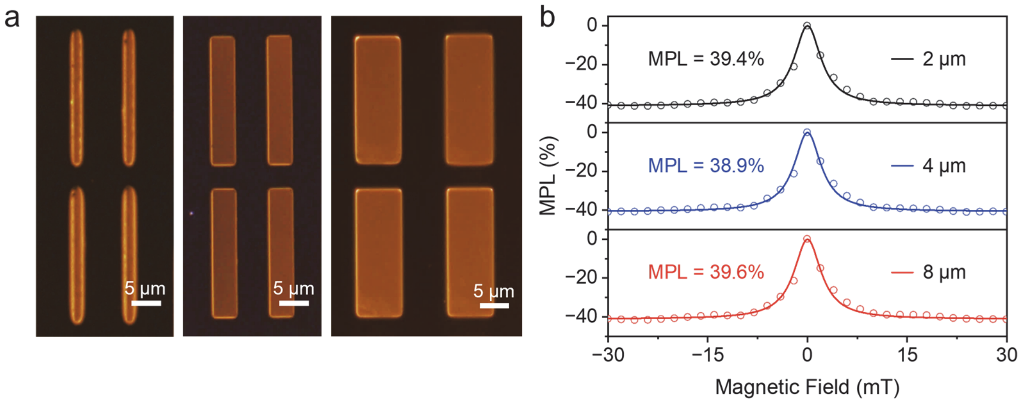

Supplementary Fig. 14 | Width dependent MPL effect in RMC. a, Fluorescence images of RMC-1.4 μm with different widths. **b,** MPL(**B**) curves measured from corresponding RMCs with different widths (2, 4 and 8 μm). The widths of RMCs (with constant thickness of 1.4 μm and length of 20 μm) are tuned from 2 to 8 μm. These samples show very similar MPL responses of ~40% at 10 mT, as the same as the length dependence. Therefore, we conclude that the MPL effect is not obviously influenced by the lateral dimensions of RMCs in our experiments. It should be note that the lateral dimensions (template-controlled) are significantly larger than the thickness (pressure-controlled) of RMCs due to the interface-confined assembly of our capillary-bridge method. In addition, the MPL effect should be more sensitive to the thickness of RMCs, since the excitation light is illuminated on the top surface and leads to exciton diffusion in the vertical direction. Source data are provided as a Source Data file.

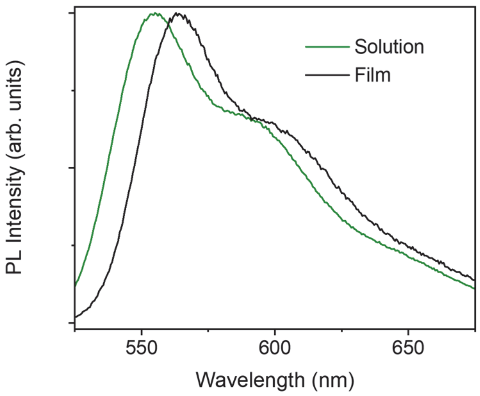

Supplementary Fig. 15 | The influence of spin conversion processes on the PL spectra in rubrene. The PL spectra for diluted rubrene solution in chloroform (green) and polycrystalline film (black). Redshift of ~8 nm from rubrene solution can be observed in the PL spectrum for polycrystalline film, which could be attribute to the contribution of spin conversion processes despite the similar shape of peaks that can be assigned to the feature of monomer PL process. Source data are provided as a Source Data file.

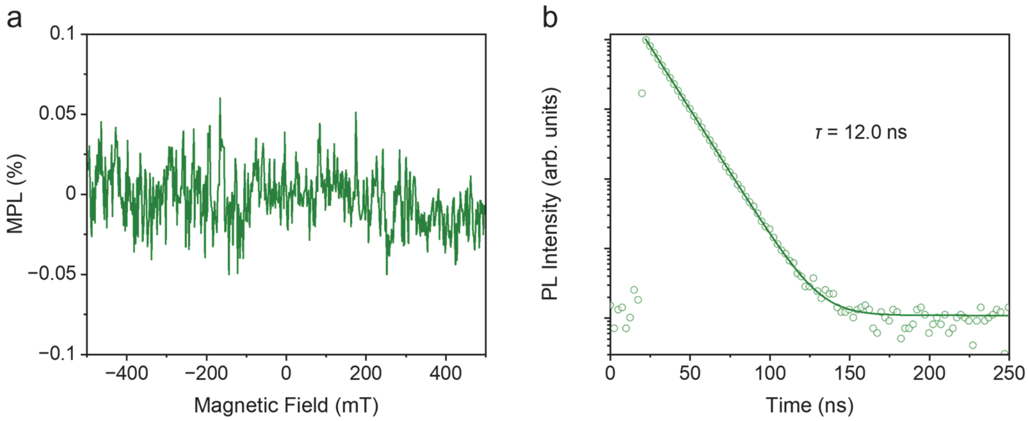

Supplementary Fig. 16 | MPL and transient PL decay curve measured from the diluted rubrene solution in chloroform. a, The absence of MPL from the spin conversion processes below the detection limit of ~0.05%. **b,** Single-exponential fitting for the excitons' lifetime of ~12.0 ns from transient PL decay which prove that there should be no spin conversion processes of SF and TF in rubrene solution. Source data are provided as a Source Data file.

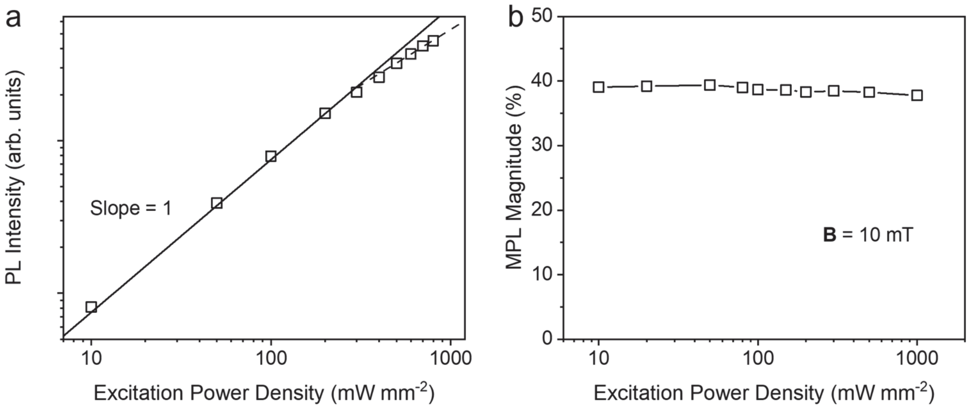

Supplementary Fig. 17 | Power dependent PL and MPL in RMC. a, PL intensity as a function of input excitation power density measured from RMC-1.4 μm. **b,** MPL magnitude at 10 mT as a function of excitation power density measured from RMC-1.4 μm. The PL emission of RMCs is monomolecular in a wide range of excitation power, and its dependence slightly deviates from the linear relation only under high excitation density. Meanwhile, the MPL magnitude slightly decreases under high excitation density, which could be attributed to the opposite MPL from non-geminate TF process. It should be mentioned that in our work all typical MPL measurements were carried out at the excitation power density of ~100 mW mm^{-2}. In this case, the non-geminate TF and its contribution to MPL should be negligible in both polycrystalline films and RMCs, and the MPL magnitude in RMCs is significantly larger than that in polycrystalline films due to the suppression of nonradiative decay in RMCs. Thus, it can be concluded that the geminate SF and TF process should be responsible for the size-dependent MPL performance in RMCs. Source data are provided as a Source Data file.

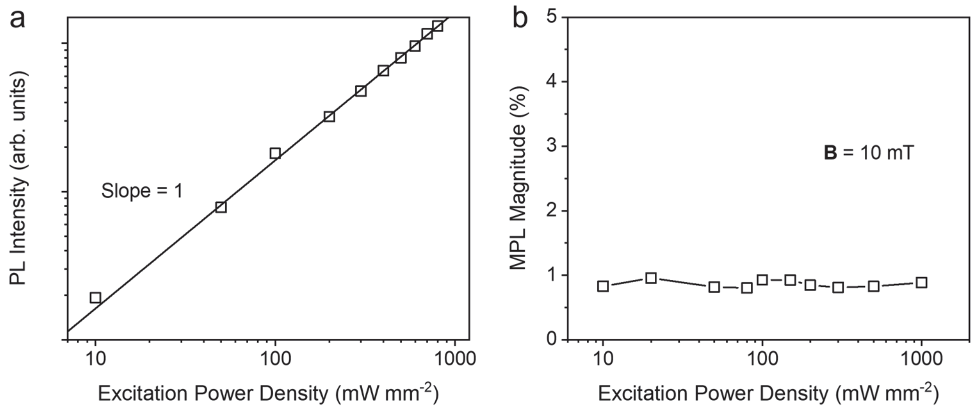

Supplementary Fig. 18 | Power dependent PL and MPL in polycrystalline rubrene. a, PL intensity as a function of input excitation power density measured from polycrystalline rubrene film. **b,** MPL magnitude at 10 mT as a function of excitation power density measured from polycrystalline rubrene film. The PL emission from polycrystalline film is dominated by the monomolecular process in the range of excitation power from 10 to 1000 mW mm^{-2}, as its power dependence is perfectly fitted with a slope of 1. It confirms the absence of non-geminate TF process due to the relatively inefficient pre-occurred SF process in polycrystalline films. Note that the MPL magnitude remains almost unchanged with the increasing of excitation power. Source data are provided as a Source Data file.

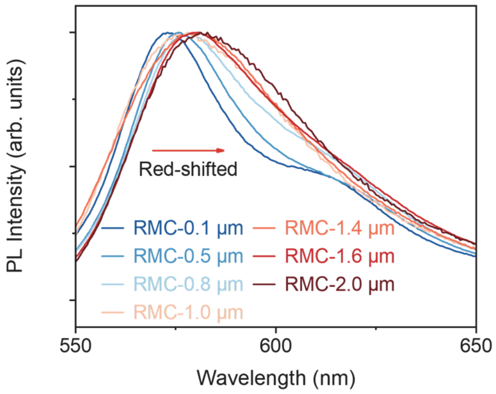

Supplementary Fig. 19 | Thickness-dependent PL spectra of RMCs. The normalized PL spectra of RMCs with increasing thickness from 0.1 to 2.0 μm. Redshift of several nanometers can be observed at the main peak around 560 nm as well as the disappearance of the side peaks at ~620 nm with increasing thickness of RMCs. Source data are provided as a Source Data file.

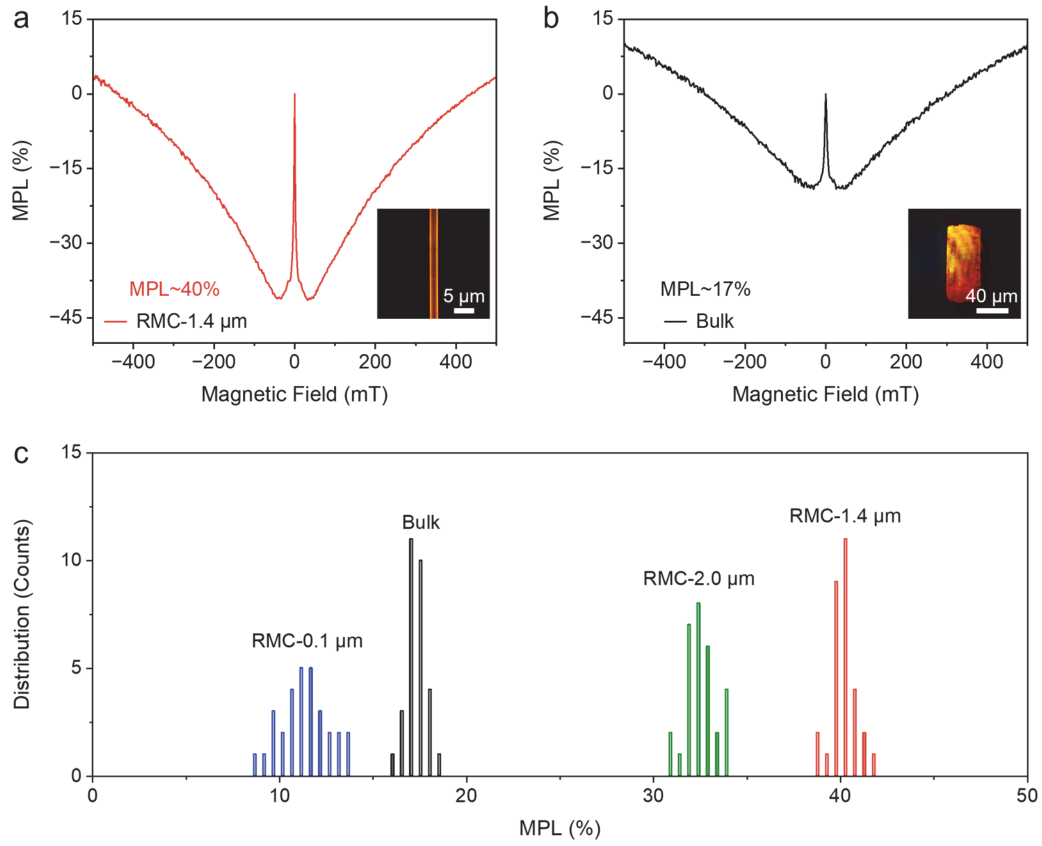

Supplementary Fig. 20 | MPL response of RMC and bulk crystal of rubrene and the statistics of MPL magnitudes. a, MPL(**B**) curves measured from RMC-1.4 μm. Inset shows the fluorescence image of RMC-1.4 μm. **b**, MPL(**B**) curves measured from bulk triclinic rubrene crystal. Inset shows the fluorescence image of the bulk rubrene crystal. **c**, Statistics of MPL magnitudes distribution measured from bulk crystals and RMCs. Source data are provided as a Source Data file.

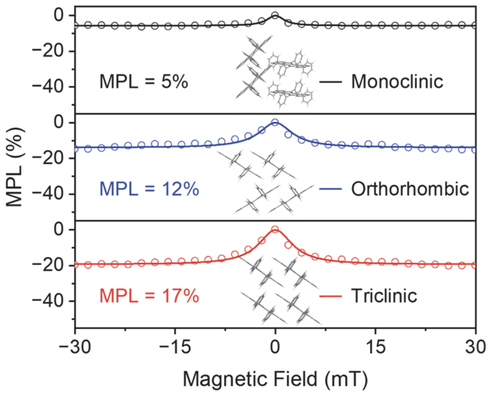

Supplementary Fig. 21 | MPL performance of rubrene in different crystal phases. MPL(**B**) curves measured from monoclinic, orthorhombic, and triclinic rubrene samples up to a field strength of 30 mT. Insets show the corresponding molecular packing styles. The MPL(**B**) curves of three rubrene crystals show the same line shape, but their MPL magnitudes vary from ~5% (monoclinic), ~12% (orthorhombic), to ~17% (triclinic) at 10 mT. The large MPL in triclinic phase could be attributed to the highly-ordered slipped-parallel packing of rubrene molecules. Source data are provided as a Source Data file.

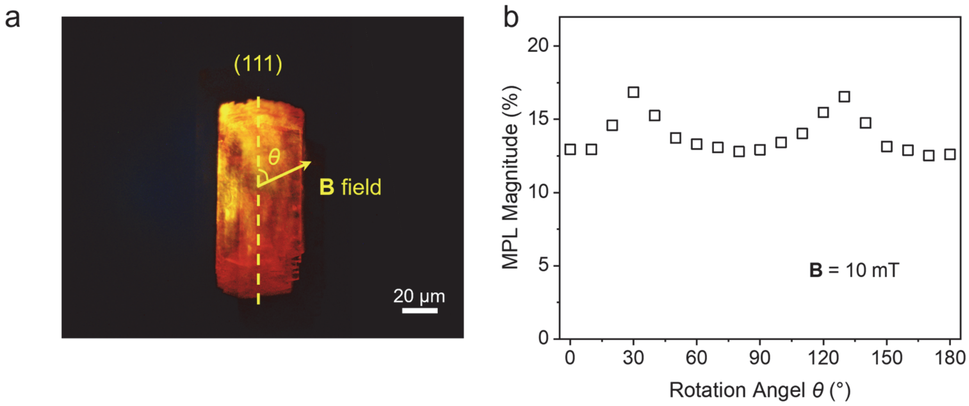

Supplementary Fig. 22 | MPL anisotropy of bulk rubrene crystal. a, Fluorescence image of single triclinic rubrene crystal. The definition of rotation angel θ according to the long axis along (111) of the crystal is shown in yellow lines. **b,** Corresponding MPL magnitude obtained at 10 mT as a function of the rotation angle of magnetic field. MPL measurements were carried out by changing the in-plane angle θ of external field relative to the long axis of rubrene crystals for bulk samples. The long axis is along the (111) direction of triclinic phase rubrene. The maximum MPL at 40° and 130° is ~17% while the minimum is ~12% at 10 mT. Source data are provided as a Source Data file.

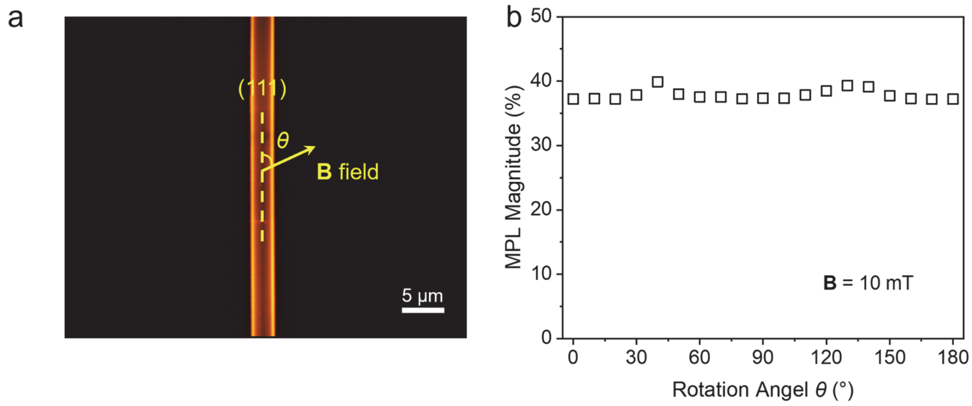

Supplementary Fig. 23 | MPL anisotropy of RMC. a, Fluorescence image of single RMC-1.4 µm. The definition of rotation angel θ according to the long axis along (111) of RMC is shown in the yellow lines. **b,** Corresponding MPL magnitude obtained at 10 mT as a function of the rotation angle of magnetic field. MPL measurements were also carried out on RMC-1.4 µm by changing the in-plane angle θ of external field relative to the long axis of RMCs. The long axis is along the (111) direction of triclinic phase rubrene. The maximum MPL appears at 40° and 130°, and the change of MPL magnitudes up on the direction of external field is ~3%. The anisotropy of MPL is observed but relatively weak compared to the overall large MPL magnitude (~40%), though the critical matching of the field direction to the resonant field does have partial contribution to the giant MPL in RMCs. Note that the MPL responses of RMCs and rubrene crystals follow the same angle dependence. Thus, the size effects of MPL observed in this work is not related to the MPL anisotropy. Source data are provided as a Source Data file.

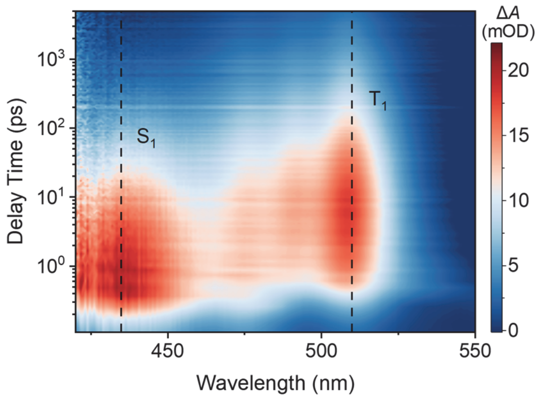

Supplementary Fig. 24 | Transient exciton dynamics in rubrene film. The pseudo-color image of time dependence transient absorption spectra of rubrene film. The feature around 440 nm can be attribute to the singlet absorption ($S_1 \rightarrow S_n$) while the feature around 510 nm can be related to the absorption of triplet state ($T_1 \rightarrow T_n$). ΔA is the change of absorption intensity. Source data are provided as a Source Data file.

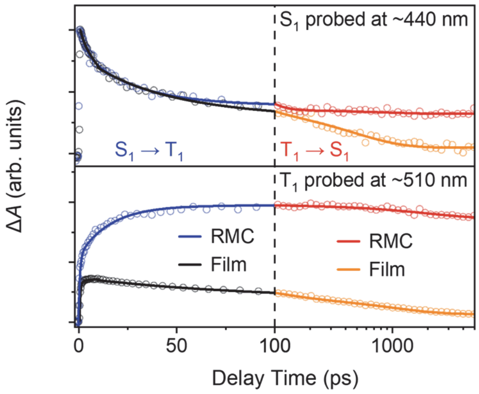

Supplementary Fig. 25 | Transient exciton kinetics in rubrene film and RMC. The transient kinetics probed at ~440 nm (S_1) and ~510 nm (T_1) in rubrene film (black line and orange line) and RMC (blue line and red line). The process can be divided into two parts where the ultrafast process before 100 ps can be attributed to the formation of T_1 from S_1 for RMC and the slow decay process in nanoseconds can be related to the decay of T_1 and the regeneration of S_1. The signal is normalized with the maximum value of Δ*A* for S_1 in rubrene film as the formation of S_1 should be only related to the excitation light under the same experimental setup. The photo-excitation induced S_1 in the upper left exhibit the similar trends in RMC and rubrene thin film that they rise to the maximum point within 1 ps and then decrease gradually. At the same time T_1 appears immediately in 1 ps and keep rising through the singlet fission process within 100 ps for RMC and within 10 ps for the thin film which is shown in the lower left of the figure. The more durable production and larger population of photo-generated T_1 in RMC indicate the enhanced singlet fission process than in the film due to the highly ordered molecular packing. As shown in the right of the figure, the radiative decay of S_1 and the reverse spin conversion of T_1 to S_1 may take control in the decay process after 100 ps according to the decreasing trends of all curves. However, both S_1 and T_1 in RMC reach the plateau and exhibit larger amount than the film due to their slow decay process. These direct observations can be attributed to the reformation of S_1 from triplet fusion in RMC, which provide us opportunity in a larger time scale to manipulate the recyclable spin conversion process of S_1 and (T_1-T_1) and to maximize the MPL effect. Source data are provided as a Source Data file.

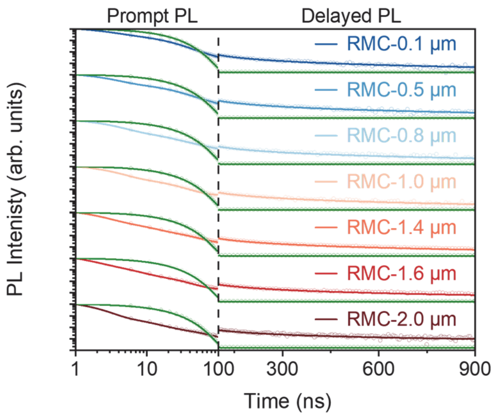

Supplementary Fig. 26 | Thickness-dependent PL decay curves in RMCs. The transient PL decay curves of RMCs with different thickness. The PL decay curves of rubrene solution (green) is also included to show the contribution of the spin conversion processes. The PL decays are decomposed into the prompt (<100 ns) and delayed (>100 ns) regimes. Note that the curves in delayed regime (>100 ns) are re-normalized for a better comparison. The PL decay of RMCs with different thickness can be fitted well with double-exponential function in the prompt region before 100 ns and with power-law function in the delayed region after 100 ns. In the first 100 ns for prompt PL, the increasing area between the decay curves of RMC with spin conversion processes and the green curves of rubrene solution without magnetic-field-sensitive spin conversion processes indicate that the efficiency of singlet fission process is enhanced with increasing thickness. Then more singlet excitons are consumed by the spin conversion process and only less excitons would be left in the direct radiative decay process which lead to the decreasing average lifetime of prompt PL from 5.81 ns in RMC-0.1 μm to 1.85 ns in RMC-2.0 μm. In the delayed PL region after 100 ns, the non-geminate triplet fusion process become slower due to the enhanced dissociation of triplet-triplet pairs, according to the decreasing value of the power function factor from 1.48 in RMC-0.1 μm to 1.09 in RMC-2.0 μm. Source data are provided as a Source Data file.

Supplementary Table 1. Fitting results of average lifetime and power-law factor for thickness-dependent PL decay.

RMC thickness / μm	Average lifetime of prompt PL (τ_{prompt}) / ns	Power-law factor of delayed PL ($m_{delayed}$)
0.1	5.81	1.48
0.5	4.42	1.45
0.8	3.54	1.43
1.0	3.15	1.42
1.4	2.52	1.37
1.6	2.23	1.28
2.0	1.85	1.09

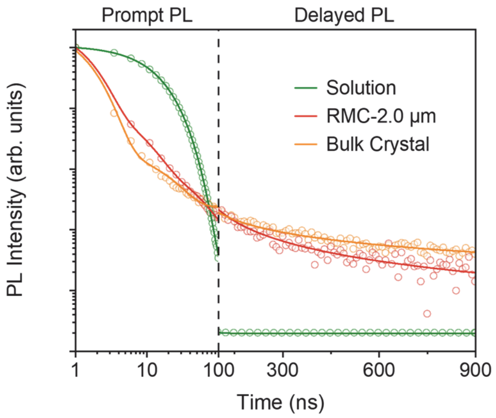

Supplementary Fig. 27 | PL dynamics in different rubrene samples. The transient PL decay curve measured from bulk rubrene crystal (orange). Transient PL decay curves measured from rubrene solution (green) and RMC-2.0 μm (red) is also included. The PL decays are decomposed into the prompt (<100 ns) and delayed (>100 ns) regimes. Note that the curves in delayed regime (>100 ns) are re-normalized for a better comparison. Further decreasing in the average lifetime (τ_{prompt} = 1.21 ns) than RMC-2.0 μm indicates the enhancing singlet fission process while the decreasing power-law factor ($m_{delayed}$ = 0.69) prove the promoted dissociation of triplet-triplet pairs and enhanced non-geminate triplet fusion process. Source data are provided as a Source Data file.

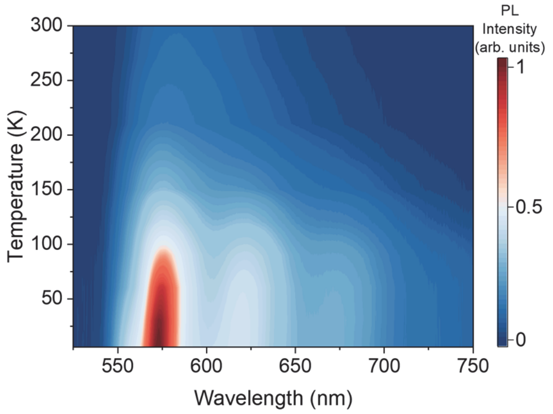

Supplementary Fig. 28 | Temperature-dependent PL emission in rubrene with spin conversion processes. The temperature-dependent PL spectra of RMC-1.4 μm. The spectra of RMC at low temperature show more vibronic features above 600 nm which can be assigned to the monomer features. Meanwhile, the PL intensity also increases for two orders of magnitude at low temperature due to the suppressed spin conversion processes. Source data are provided as a Source Data file.

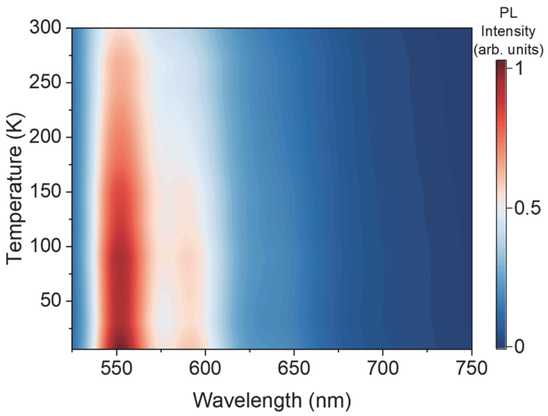

Supplementary Fig. 29 | Temperature-dependent PL emission in rubrene without spin conversion processes. The temperature-dependent PL spectra of monomer dispersed rubrene in polymethyl methacrylate (PMMA). The monomer rubrene dispersed by PMMA keeps the vibronic features when cooling down and the PL intensity increases for only two times due to the suppressed vibrational relaxation instead of reduced spin conversion processes of singlet fission and triplet fusion at cryogenic temperatures. Source data are provided as a Source Data file.

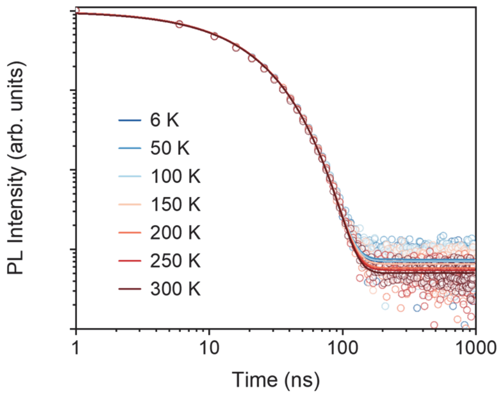

Supplementary Fig. 30 | Temperature-dependent PL decay curves in monomer rubrene. The transient PL decay curves measured from monomer dispersed rubrene in PMMA at different temperature from 6 K to 300 K. The monomolecular rubrene dispersed by PMMA shows the similar single-exponential lifetime of ~16 ns at different temperature. Source data are provided as a Source Data file.

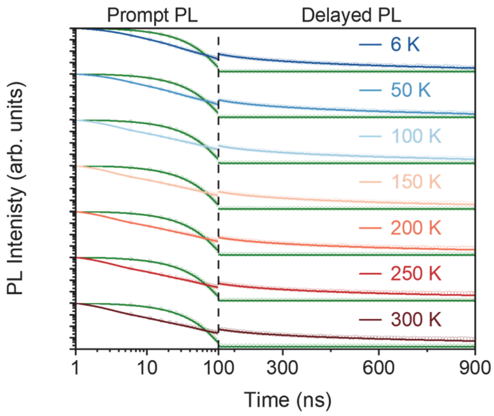

Supplementary Fig. 31 | Temperature-dependent PL decay curves in RMCs. The transient PL decay curves measured from RMC-1.4 μm at different temperature. The PL decay curves of rubrene solution (green) is also included to show the contribution of the spin conversion processes. The PL decays are decomposed into the prompt (<100 ns) and delayed (>100 ns) regimes. Note that the curves in delayed regime (>100 ns) are re-normalized for a better comparison. The PL decay of RMC-1.4 μm measured at different temperatures are fitted with double-exponential function in the prompt region before 100 ns and with power-law function in the delayed region after 100 ns. In the prompt PL region, the increasing area between the decay curves of RMC and rubrene solution and the decreasing average lifetime of prompt PL with rising temperature indicate the enhanced thermal activated singlet fission process. In the delayed region after 100 ns, the non-geminate triplet fusion process is slower with increasing temperature due to the dissociation of triplet-triplet pairs, proved by the decreasing power-law factor for delayed PL. The temperature-dependent results are quite similar to the thickness-dependent results as the rising temperature play the same role in the spin conversion process as growing thickness. Source data are provided as a Source Data file.

Supplementary Table 2. Fitting results of average lifetime and power-law factor for temperature-dependent PL decay.

Temperature / K	Average lifetime of prompt PL (τ_{prompt}) / ns	Power-law factor of delayed PL ($m_{delayed}$)
6	4.50	1.71
50	3.90	1.69
100	3.01	1.64
150	2.73	1.58
200	2.64	1.51
250	2.58	1.45
300	2.51	1.43

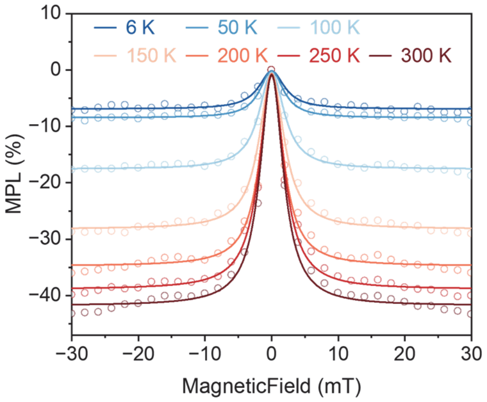

Supplementary Fig. 32 | Temperature-dependent MPL response of RMC. The MPL(**B**) curves measured from RMC-1.4 μm. The MPL magnitude increases with the temperature as the singlet fission process is thermally activated. Source data are provided as a Source Data file.

Supplementary references

1. Yago, T., Ishikawa, K., Katoh, R. & Wakasa, M. Magnetic Field Effects on Triplet Pair Generated by Singlet Fission in an Organic Crystal: Application of Radical Pair Model to Triplet Pair. *J. Phys. Chem. C* **120**, 27858-27870 (2016).

2. Wang, M. S. et al. Exploring mechanisms for generating spin-orbital coupling through donor–acceptor design to realize spin flipping in thermally activated delayed fluorescence. *J. Mater. Chem. C* **8**, 3395-3401 (2020).

3. Wang, M. S. et al. Identifying Different Spin Mixing Channels Occurring in Charge-Transfer States. *J. Phys. Chem. C* **124,** 14832-14837 (2020).

4. He, L., Li, M. X. Urbas, A. & Hu, B. Magnetophotoluminescence line-shape narrowing through interactions between excited states in organic semiconducting materials. *Phys. Rev. B* **89**, 155304 (2014).

5. Deotare, P. B. et al. Nanoscale transport of charge-transfer states in organic donor–acceptor blends. *Nat. Mater.* **14**, 1130-1134 (2015).

6. Johnson, R. C. & Merrifield, R. E. Effects of magnetic fields on mutual annihilation of triplet excitons in anthracene crystals. *Phys. Rev. B* **1**, 896-902 (1970).

7. Merrifield, R. E., Avakian, P. & Groff, R. P. Fission of singlet excitons into pairs of triplet excitons in tetracene crystals. *Chem. Phys. Lett.* **3**, 386-388 (1969).

8. Hodges, M. P. P., Grell, M., Morley, N. A. & Allwood, D. A. Wide Field Magnetic Luminescence Imaging. *Adv. Funct. Mater.* **27**, 160661 (2017).

9. Perkinson, C. F. et al. Magnetic-Field-Switchable Laser via Optical Pumping of Rubrene. *Adv. Mat.* **34**, 2103870 (2022).

10. Basel, T. et al. Magnetic Field Enhancement of Organic Light-Emitting Diodes Based on Electron Donor-Acceptor Exciplex. *Adv. Electron. Mater.* **2**, 1500248 (2016).

11. Yago, T. et al. Triplet-Triplet Annihilation via the Triplet Channel in Crystalline 9,10-Diphenylanthracene. *J. Phys. Chem. Lett.* **13**, 8768-8774 (2022).

12. Nagata, R., Nakanotani, H., Potscavage, W. J. & Adachi, C. Exploiting Singlet Fission in Organic Light-Emitting Diodes. *Adv. Mater.* **30**, 18014 (2018).

13. De, R., Fujiwara, Y., Haino, T. & Tanimoto, Y. Effect of high magnetic fields on intramolecular exciplex fluorescence of pyrene and dimethylaniline systems. *Chem. Phys. Lett.* **315**, 383-389 (1999).

14. Buck, J. T. & Mani, T. Magnetic Control of Recombination Fluorescence and Tunability by Modulation of Radical Pair Energies in Rigid Donor-Bridge-Acceptor Systems. *J. Am. Chem. Soc.* **142**, 20691-20700 (2020).

15. Katoh, R. et al. Singlet Fission in Fluorinated Diphenylhexatrienes. *J. Phys. Chem. C* **121**, 25666-25671 (2017).
